# Evolutionary Perspective and Expression Analysis of Intronless Genes Highlight the Conservation of Their Regulatory Role

**DOI:** 10.3389/fgene.2021.654256

**Published:** 2021-07-09

**Authors:** Katia Aviña-Padilla, José Antonio Ramírez-Rafael, Gabriel Emilio Herrera-Oropeza, Vijaykumar Yogesh Muley, Dulce I. Valdivia, Erik Díaz-Valenzuela, Andrés García-García, Alfredo Varela-Echavarría, Maribel Hernández-Rosales

**Affiliations:** ^1^Instituto de Neurobiología, Universidad Nacional Autónoma de México, Querétaro, Mexico; ^2^Centro de Investigacioìn y de Estudios Avanzados del IPN, Unidad Irapuato, Guanajuato, Mexico; ^3^Centro de Física Aplicada y Tecnología Avanzada, Universidad Nacional Autónoma de México, Querétaro, Mexico; ^4^Centre for Developmental Neurobiology, Institute of Psychiatry, Psychology, and Neuroscience, King’s College London, London, United Kingdom

**Keywords:** intronless genes, exon-intron architecture, embryonic telencephalon, protocadherins, histones, transcription factors, microproteins, evolutionary histories

## Abstract

The structure of eukaryotic genes is generally a combination of exons interrupted by intragenic non-coding DNA regions (introns) removed by RNA splicing to generate the mature mRNA. A fraction of genes, however, comprise a single coding exon with introns in their untranslated regions or are intronless genes (IGs), lacking introns entirely. The latter code for essential proteins involved in development, growth, and cell proliferation and their expression has been proposed to be highly specialized for neuro-specific functions and linked to cancer, neuropathies, and developmental disorders. The abundant presence of introns in eukaryotic genomes is pivotal for the precise control of gene expression. Notwithstanding, IGs exempting splicing events entail a higher transcriptional fidelity, making them even more valuable for regulatory roles. This work aimed to infer the functional role and evolutionary history of IGs centered on the mouse genome. IGs consist of a subgroup of genes with one exon including coding genes, non-coding genes, and pseudogenes, which conform approximately 6% of a total of 21,527 genes. To understand their prevalence, biological relevance, and evolution, we identified and studied 1,116 IG functional proteins validating their differential expression in transcriptomic data of embryonic mouse telencephalon. Our results showed that overall expression levels of IGs are lower than those of MEGs. However, strongly up-regulated IGs include transcription factors (TFs) such as the class 3 of POU (HMG Box), *Neurog1, Olig1*, and *BHLHe22, BHLHe23*, among other essential genes including the β-cluster of protocadherins. Most striking was the finding that IG-encoded *BHLH* TFs fit the criteria to be classified as microproteins. Finally, predicted protein orthologs in other six genomes confirmed high conservation of IGs associated with regulating neural processes and with chromatin organization and epigenetic regulation in *Vertebrata*. Moreover, this study highlights that IGs are essential modulators of regulatory processes, such as the Wnt signaling pathway and biological processes as pivotal as sensory organ developing at a transcriptional and post-translational level. Overall, our results suggest that IG proteins have specialized, prevalent, and unique biological roles and that functional divergence between IGs and MEGs is likely to be the result of specific evolutionary constraints.

## Introduction

**Most euk**aryotic genes contain introns, nucleotide DNA sequences that after transcription as part of the messenger RNA are removed by splicing during its maturation. Since the introns interrupt the multiple exonic sequences, these genes are thus termed multiple exon genes (MEGs). Eukaryotic genomes, however, also contain an important proportion of genes in which the coding sequence is contained within a single exon. Diverse studies of genes of this type have been performed over the past decades and have been variously referred to as “single-exon genes” (SEGs) and “intronless genes” (IGs), both terms carrying some ambiguity as genes containing an intron in their 5**′ UTR are often** included among them (Sunahara et al., 1990; Gentles and Karlin, 1999; Sakharkar et al., 2002, 2005a, 2006; Tine et al., 2011; Zou et al., 2011; Yan et al., 2014). For example, a recent ontology defines SEGs as nuclear genes with functional protein-coding capacity whose coding sequence comprises only one exon, thus including genes with introns in their untranslated regions termed uiSEGs, as well as those lacking introns entirely, termed “*Intronless Genes”* (Jorquera et al., 2018). Pseudogenes, functional RNAs, tRNA, rRNA, ribozyme long non-coding RNAs, and miRNAs are excluded from this definition. To avoid any possible ambiguity, in this article we use the term “Intronless genes” in the narrow definition of Jorquera et al. (2018) as protein-coding nuclear genes completely devoid of introns.

Owing to their prokaryotic-like architecture, IGs in eukaryotic genomes, provide interesting datasets for computational analysis in comparative genomics and for the study of evolutionary trajectories. Comparative analysis of their sequences in different genomes could allow the identification of their unique and conserved features, thus providing insights into the role of introns in gene evolution leading to a better understanding of genome architecture and arrangement.

The abundant presence of introns in most genes of multicellular organisms entails regulatory processes associated with the generation of multiple splice variants missing in intronless genes. The absence of splicing events in IGs represents a higher transcriptional fidelity, making them even more valuable for regulatory roles. To date, more than 2000 genes with a single coding exon in the human genome have been classified (Jorquera et al., 2016). Among them, a considerable fraction of IGs encode G-protein-coupled receptors (GPCRs), core canonical histones which are integral part of nucleosomes and often confer specific structural and functional features, transcription factors, proteins involved in signal transduction, regulation of development, growth, and cell proliferation (Sakharkar et al., 2005a; Grzybowska, 2012).

The expression of IGs has been proposed to be highly specialized for neural functions and linked to diseases such as cancer, neuropathies, and developmental disorders. Examples of IGs with clinical relevance are the *RPRM* gene related to gastric cancer which causes increased cell proliferation and possesses tumor suppression activity (Amigo et al., 2018) and the protein kinase *CK2*α gene which is up-regulated in all human cancers (Hung et al., 2010). Other IGs linked to cancer include *CLDN8* in colorectal carcinoma and renal cell tumors, *ARLTS1* in melanoma, and *PURA* and *TAL2* in leukemia (Grzybowska, 2012). IGs have also been associated with neuropathies, such as *ECDR1*, a cerebellar degeneration-related protein, and *NPBWR2*, a neuropeptide B/W receptor type (Louhichi et al., 2011).

Regarding their role in the diseases described above, IGs in humans are potential clinical biomarkers and drug targets that deserve careful consideration (Ohki et al., 2000; Grzybowska, 2012; Liu et al., 2017). Their functional role and their evolutionary conservation in other genomes, however, remains poorly understood. Furthermore, there is a current debate between related theories that place the origin of introns, early or late during the evolutionary history of eukaryotes (de Souza, 2003; Fedorova and Fedorov, 2003).

With this backdrop, our work aimed to characterize the functional role of mouse IGs and to infer their evolutionary pattern across six additional vertebrate genomes. We have analyzed their expression, particularly during brain development at early embryonic stages, and their potential as transcriptional as well as post-translational modulators.

Overall, this study sheds light on the concerted role played by this peculiar group of genes and helps contrast the functional features of intron-containing and intronless genes across vertebrate species and their collective evolutionary roadmaps.

## Materials and Methods

### Data Extraction and Curation for MEGs and IGs

**FIGURE 1:**
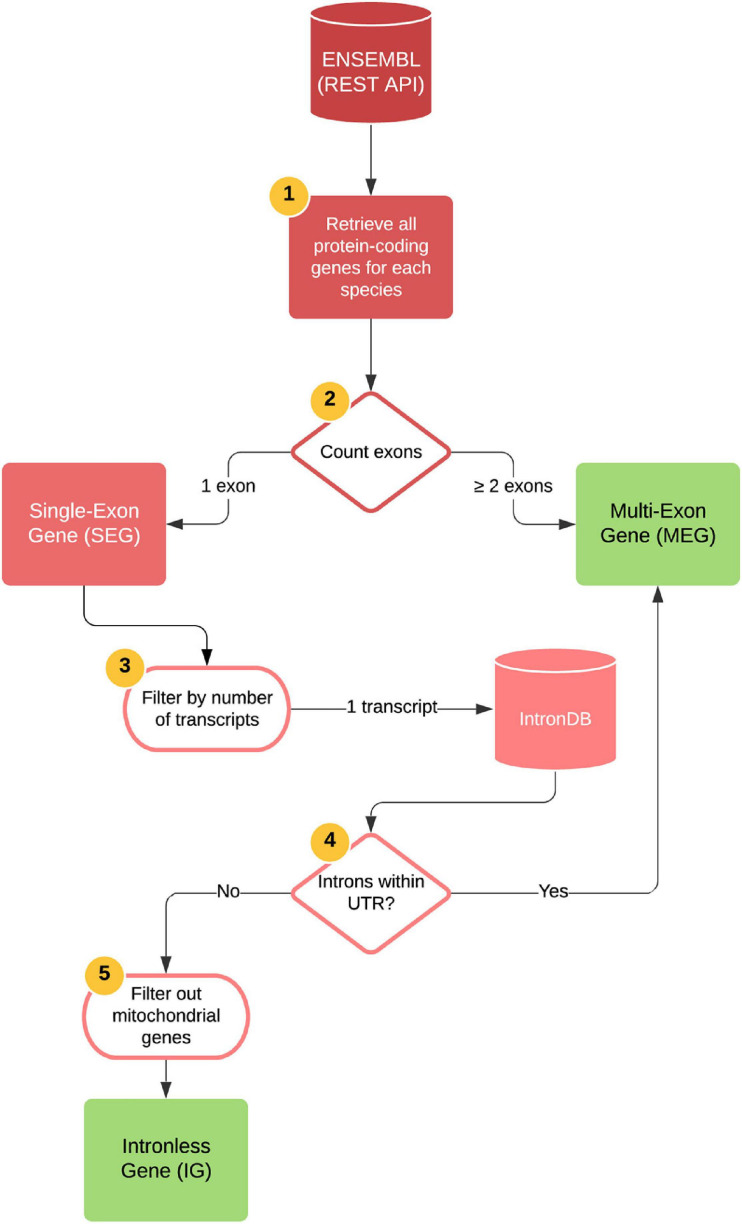
Bioinformatics pipeline for generating IG and MEG datasets. Automatized steps (represented as 1–4): all protein-coding genes were retrieved for each species by accessing the Ensembl REST API platform. The number of exons for each gene were counted, and genes with two or more exons were identified as MEGs. Genes with one single exon were identified as SEGs and were subsequently filtered by their number of transcripts, keeping only those with one transcript. Using the IntronDB, we then classified these genes according to the presence of introns within their UTRs. Genes with introns within these regions were identified as MEGs and those without, as IGs. Manual curation was performed to filter out mitochondrial genes and genes with incomplete protein annotations (step 5). Output files are depicted as green squares.

Data were extracted using Python scripts^1^ and Ensembl APIs. Seven vertebrate genomes including *Mus musculus, Homo sapiens, Pan troglodytes, Monodelphis domestica, Rattus norvegicus, Gallus gallus*, and *Danio rerio* assembled at a chromosome level were accessed at the Ensembl REST API platform^2^ (using Python with the ensembl_rest package). For an explanation of the species choice see section “*Search for Orthologs of Mouse IGs.*” The pipeline process was as follows: protein-coding genes with CDS identifiers for transcripts for all chromosomes were retrieved and classified into a temporary dataset that contained genes with a single coding exon (temp-DS1) and a dataset containing “multiple exon genes” (MEGs) depending on exon and transcript count (Figure 1). The former was then submitted to the Intron DB^3^ to filter out genes with UTR introns. The output of the pipeline was a second temporary dataset containing protein-coding genes that did not contain introns in their entire length (temp-DS2). After data extraction, a manual curation step was performed to assess their nuclear nature and protein-coding transcript biotype, which allowed us to discard proteins encoded in the mitochondrial genome, hence yielding the final dataset containing only protein-coding nuclear genes completely devoid of introns, or “Intronless genes” (IG) (Figure 1). The final MEG dataset contained 20,694 protein-coding genes with two or more exons and the IG dataset contained 1,116 protein-coding genes with only one exon and one transcript.

### Computational Prediction of Mouse Intronless Gene Function

The mouse IG and MEG datasets were used to perform an over-representation analysis of functional assignment using the following databases: SUPERFAMILY^4^ (proteins of known three-dimensional structure); Pfam^5^ (protein domains); and PROSITE^6^ (biologically meaningful signatures or motifs). All tests used MEGs as controls to determine unique, shared or overrepresented features among both types of genes. For data visualization of SUPERFAMILY and Pfam results we employed the ClusterProfiler R package (Yu et al., 2012), and Python scripts using a hypergeometric test for PROSITE enrichment.

### Functional Enrichment Analysis of IG and MEG Proteins

The functional enrichment analyses were performed using Metascape^7^ for the biological process category, including KEGG and Reactome pathways. First, the functional enrichment of the 1,116 mouse IG proteins was performed using all mouse proteins as a background “universe” (selecting input as species: *M. musculus*, analysis as species: *M. musculus*). Then, in a second approach the meta-analysis workflow was used to compare enriched terms for the list of orthologs of mouse IGs regarding their grouping into five *“ages,”* each of them corresponding to one of the taxonomic categories: *Vertebrata*, *Tetrapoda*, *Theria*, *Eutheria*, and *Muridae*.

Out of the 1,116 IGs, 543 have orthologs across the analyzed species. Mouse IG orthologs that conserved the IG structure were compared against MEGs conserved as MEGs in other organisms. The meta-analysis workflow was used to compare enriched terms for the list of mouse orthologs in the aforementioned genomes to the pathways of three random samples of the same size of multi-exon genes to confirm that we obtained similar results. For this analysis, orthologs were clustered regarding their previously inferred *“age”* in five groups (selecting input as species: any species, analysis as species: *M. musculus*).

Finally, we performed a third approach to determine the conservation of the functional role of IGs. First, we determined the overrepresented GO terms for biological processes and molecular function of the orthologs from the seven genomes using AmiGO2^8^ were obtained. Then, GO terms with their corresponding *p-*values were clusterized using REVIGO which finds a representative subset of the terms using an algorithm that relies on semantic similarity measures (Supek et al., 2011).

### Data Source and Differential Expression Analysis

Read counts from a previous transcriptomic analysis of mouse embryonic telencephalon were used to identify differentially expressed genes (Muley et al., 2020). The transcriptomes were obtained using the Illumina HiSeq RNA sequencing (RNA-seq) platform. The procedure for read-counts normalization, and to calculate differential expression analysis is described in https://data.mendeley.com/datasets/rdt5757cbw/1. A gene was considered expressed if its count-per-million (CPM) value was above 5.66-7. Mouse IG and MEG datasets were submitted to analysis to determine the directionality of the change in expression at developmental stage A (E.9.5) compared to stage B (E.10.5). Genes having significant *p*-values with positive log2–fold change represent an increased expression (UP), those with negative log2-fold change are considered with decreased expression (DN), while gene expression with *p*-values above 0.05 represents no change between stages (NC), and read-count lower than five in less than four samples out of eight are considered not expressed (NE).

### Functional Enrichment Analysis of Differentially Expressed IGs and MEGs

The functional enrichment was assessed using the over-representation analysis of the functional assignment. Genes with differential expression up to two log2-fold change values were considered as up-regulated with a *p*- and *q*-value set at 0.05 and 0.10, respectively. The ClusterProfiler R package (Yu et al., 2012) was employed for data analysis and visualization.

### Post-translational Modifications and Regulatory Assignment of IG Proteins

For post-translational modification assignments of IG and MEG proteins, the dbPTM^9^ was used. A two proportion *Z*-test was used to assess whether the proportions of each post-translational modification among IG and MEG proteins were similar. The *p*-value was set at 0.05. When the resulting *p*-value was not significant, meaning that the proportions of IG and MEG proteins were similar for a specific post-translational modification, this was classified as “similar.” On the other hand, when the resulting *p*-value was <0.05 the post-translational modification was classified as more abundant in ‘‘IG’’ or ‘‘MEG’’ depending on which one had a higher relative percentage of such modification. Post-translational modifications exclusive of either IG or MEG proteins were classified as ‘‘unique.’’ Then, using the miPFinder program^10^, we determined the mouse gene candidates for IG-encoding microproteins.

### Search for Orthologs of Mouse IGs

Mouse peptide sequences were submitted to Proteinortho (Lechner et al., 2011, 2014) to infer orthologous genes in the genomes of rat, human, chimp, opossum, chicken, and zebrafish. As a first step, Proteinortho performs sequence comparison between each pair of genomes and reports best bidirectional hits (BBHs) for alignments with equal or above fifty percent of sequence identity. In a second step, it represents each gene or protein as a node of a graph and places an edge between two genes if they were identified as a BBH, then, it applies a clustering algorithm and finally reports orthogroups and the orthology relations as pairs of genes in two different genomes.

Each of the species used in this study is a model organism of a different taxonomic level, and therefore, the conservation of mouse orthologs in close or distant related species reveals the “age” of the gene. The conservation was measured by gradually including more species, and the resulting groups were labeled with the name of the largest taxonomical category that includes all species within a group. Therefore, for orthologs of mouse IG genes that were identified in rat, they are said to be conserved in *Muridae;* those present in *Muridae*, in human, and in chimp are said to be conserved in the group *Eutheria;* those found in all the previous species and in opossum as well are conserved in the group *Theria;* those also present in chick are conserved in *Tetrapoda*; and finally, those also conserved in zebrafish are conserved in *Vertebrata*. This classification, however, is only used to refer to the conservation among the species analyzed in the present study.

### Reconstruction of the Evolutionary History of Mouse IGs and Their Conservation in Other Organisms

From the ProteinOrtho predictions, orthology graphs were constructed, and an in-house developed method for the evolutionary reconstruction of gene families was used. This method implements the theory reported in Hernandez-Rosales et al. (2012) and Hellmuth et al. (2013), and it can be found at https://gitlab.com/jarr.tecn/revolutionh-tl. This tool starts by performing a modular decomposition (Tedder et al., 2008) on orthology graphs and then inferring the corresponding gene trees. Each internal node of these trees represents an evolutionary event: duplication or speciation. Subsequently, the gene trees are reconciled with the species tree to determine in which branch of the species tree duplication events occur and, at the same time, infer gene losses. This method allows us to infer how ancestral a gene is, determined by its orthologs in the other species, as well as to identify species-specific genes. Finally, we identified the orthologs of the 1,116 mouse IG proteins that were conserved as IGs, or that were identified as MEGs in the abovementioned genomes.

### Syntenic Conservation of the β-Protocadherin Cluster

To determine the syntenic conservation of the mouse protocadherin IG members of the beta cluster across the selected genomes, the genomic coordinates of orthologs genes were retrieved from GTF files employing custom R scripts and plotted using the genoPlotR R package.

## Results

### Functional Assignment of Protein Coding IGs in the Mouse Genome

The origin of IGs has been explained mostly by retrotransposition, which occurs by homologous recombination between the genomic copy of a gene and an intronless cDNA (Kaessmann et al., 2009). Mouse IGs represent 6% of the total number of one-exon genes, while retrotransposed single-exon pseudogenes with lost molecular function constitute almost half of them (coding, non-coding, pseudogenes) (Supplementary Figure 1).

Computational analysis was performed to identify mouse IGs. Then, based on the comparative annotation of IG and MEG datasets, a study was performed to identify their unique and shared molecular and biological features.

The grouping of IGs by protein domains that have an evolutionary relationship (SUPERFAMILY database) revealed a higher enrichment of the histone fold, 4-helical cytokine family of signal transducers, and transcription factor families including the Poxvirus and Zinc finger (POZ) domain, “Winged helix” DNA-binding domain, High Mobility Box group (HMG-box), and A DNA-binding domain in eukaryotes, as well as the transmembrane protein families Cadherin-like, and Frizzled cysteine-rich domain. MEG-encoded proteins, in contrast, are enriched in protein kinase-like, immunoglobulin, Krüppel associated box (KRAB) domain, and Armadillo repeat motifs (ARM) repeat families. The top enriched structural families of IG and MEG groups are shown in Supplementary Figure 2.

**FIGURE 2:**
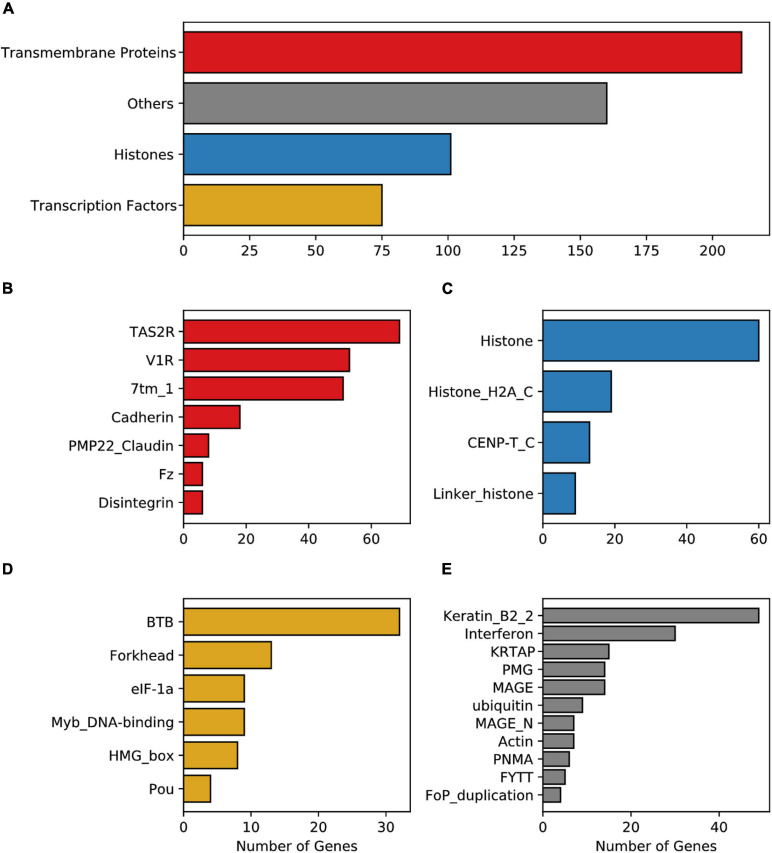
Enrichment of the Pfam protein domains in mouse IG proteins. **(A)** Enriched domains of proteins grouped into four main classes, transmembrane proteins (red), histones (blue), transcription factors (yellow), and others (gray). Enrichment of domain terms was calculated with a significant background list associated with the Pfam domain and p-adjusted values above 0.05. **(B)** Pfam domains grouped in the *transmembrane proteins* class, **(C)** Pfam domains grouped in the *histone* class, **(D)** Pfam domains grouped in the *transcription factors* class, **(E)** Pfam domains considered as *others*.

The analysis of the conserved functional domains (Pfam database) among the enriched protein families encoded by mouse IGs, revealed 598 hits of three main classes: 253 were transmembrane protein receptors, 101 core histones, 84 transcription factors, and 160 that belong to other classes (Figure 2A). Among the transmembrane protein receptors, the most enriched domains were Taste 2 receptors (TAS2R), Vomer-nasal 1 receptors (V1R), and seven transmembrane group 1 (7tm_1), common in GPCR and vomeronasal receptors (Figure 2B). Other domains identified were cadherin, Pheripheral myelin protein 22 (PMP22-claudin), (Desintegrin and metalloproteinase domain (ADAM), Disintegrin, and Frizzled (FZ). In the transcription factor group, Broad-Complex, tramtrack, and bric- -brac (BTB), Myb DNA-binding, HMG-Box, and forkhead were enriched protein domains in the mouse IGs compared to MEGs (Figure 2C). Meanwhile, in the histone group, four domains were observed: Histone, Histone H2A 1363 C-terminal (H2AC), Centromere kinetochore component CENP-T histone (CENPTC), and Linker histone (Figure 2D). Finally, in the other groups we found among others Keratin, Interferon, Ubiquitin, Actin, and FYTT enriched domains (Figure 2E). The classification of IGs in functional groups mentioned above was then used for the transcriptional analysis (Figure 2).

**FIGURE 3:**
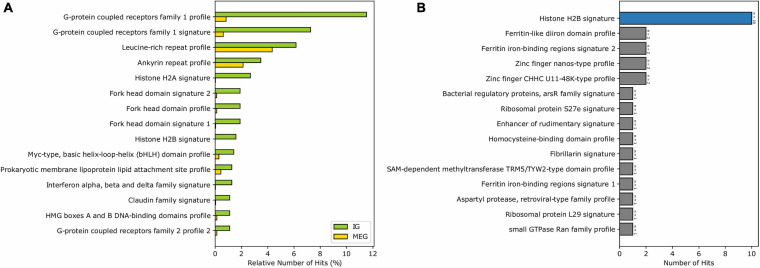
Enrichment of the PROSITE signatures in mouse IG proteins compared to MEG proteins. **(A)** The 15 most abundant PROSITE signatures of IG proteins. **(B)** PROSITE signatures exclusive of IG proteins (signatures belonging to the *Histones* class are shown in blue).

Analysis of biologically significant motifs (PROSITE database) among MEG and IG proteins identified a total of 1,239 (12,546 hits) and 144 (634 hits) distinct protein signatures, respectively. Interestingly, among the most abundant motifs in the mouse IGs are GPCR, leucine-rich repeat, histone, transcription factor Forkhead domain, Myc-type basic helix-loop-helix (bhlh) motifs, ankyrins, and cadherin domains which were found to be infrequent in MEG proteins (Figure 3A). It is noteworthy, however, that among the top motifs that were unique to IG proteins H2B signature was the largest group (Figure 3B). Hence, these results show that most of the top predictions of IGs signatures are characteristic of transmembrane receptors, histones, and specific transcription factors, having a unique signature for histones.

**FIGURE 4:**
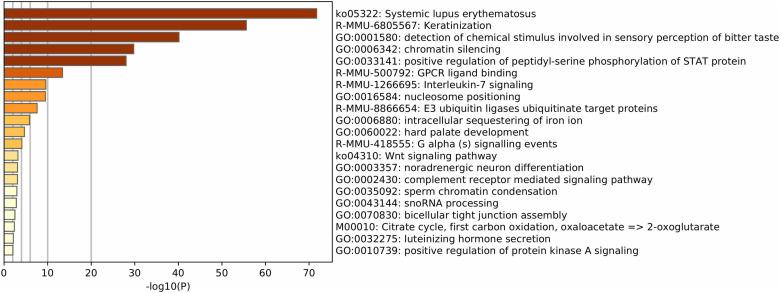
Functional enrichment of mouse IG proteins. Gene ontology enrichment and pathways for IG proteins. The color key from yellow to brown indicates high to low *p*-values, respectively.

Finally, the functional enrichment of mouse IGs revealed biological pathways associated with genetic and protein regulatory processes including detection of chemical stimulus involved in sensory perception of the bitter taste, chromatin silencing, positive regulation of peptidyl-serine phosphorylation of STAT proteins, and nucleosome positioning (–log10–34.23 > −3.27). Other functions detected were immune, neuro-specific, and development processes such as mmu05322-Systemic lupus erythematosus, R-MMU-6805567 Keratinization, R-MMU-500792 GPCR ligand binding, R-MMU-1266695 Interleukin 7 signaling, hard palate development, and noradrenergic neuron differentiation (–log10–29.94 > −3.75) (Figure 4).

Altogether, functional assignment analysis suggests that IGs have distinct biological roles in comparison to MEGs.

### Up-Regulation of IGs Reveal Their Regulatory Role on Neural Functions Through Mouse Development

Previous studies detected enrichment of neural-related functions among IGs (Grzybowska, 2012). Moreover, since the expression of MEGs is modulated by the balance between the rate of transcription elongation and the alternative splicing of exons (Fong et al., 2014), we hypothesized that the natural absence of splicing on IG mRNAs could confer them differential regulatory roles in complex biological processes. Therefore, it was our interest to identify and analyze IGs that are expressed in mice during brain development. For that purpose, we analyzed expression data from the developing mouse telencephalon at stages in which its patterning is taking place (E9.5 and E10.5) (Muley et al., 2020).

**FIGURE 5:**
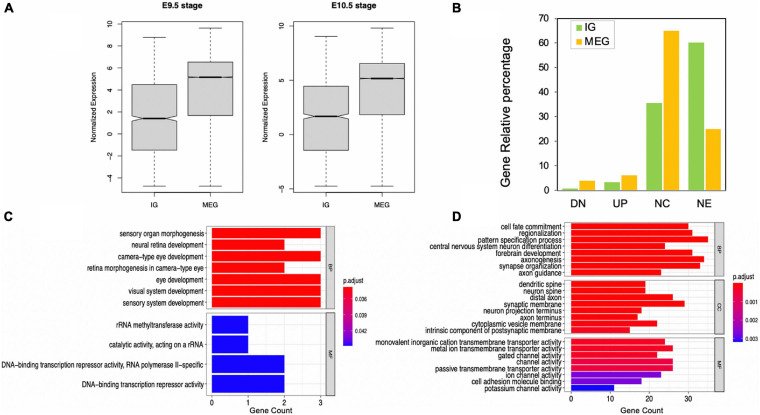
Expression levels of IGs in mouse embryonic telencephalon compared to MEGs. **(A)** IG and MEG normalized expression at 9.5 and 10.5 embryonic stages; **(B)** IG and MEG, gene differential expression groups in 9.5 and 10.5 stages, (UP) upregulated, (DN) downregulated, (NC) no expression changes, (NE) not expressed. Enrichment of functions in upregulated IGs **(C)** and MEGs **(D)**. Each barplot shows significantly enriched gene ontology (GO) terms on the y-axis, colored by their *p*-adjusted values, while gene count is represented on the x-axis. Test gene sets for enrichment analysis were the up-regulated genes in each dataset, and the background set was all up-regulated genes. GO terms are grouped by BP (biological process), CC (cellular component), and MF (molecular function).

Overall, the expression of IGs was lower than that of MEGs (Figure 5A), which is consistent with previous *in silico* observations (Sakharkar et al., 2005a). Out of 1,116 transcripts, differential expression analysis was performed for 1,087, with 37 of them (3.4%) showing up-regulation and nine down-regulation (0.82%) from gestational day E9.5 to E10.5 Moreover, 387 (35.63%) did not change expression, and 653 (60.12%) were not expressed during the analyzed stages (Figure 5B). Meanwhile, among MEGs, 1247 were up-regulated (6.13%), 789 were down-regulated (3.88%), 13,198 had no expression changes (64.93%), and 5090 did not show expression (25.04%) (Figure 5B). It is noteworthy that an inverse expression pattern of genes with no expression changes (a higher percentage of MEGs than of IGs) and those not expressed (a higher percentage of IGs than of MEGs) was found in this comparison.

Our analysis revealed that all up-regulated IGs are exclusively enriched in biological pathways in eye and sensory organ development processes compared to MEGs also involved in other developmental and neural function pathways (Figures 5C,D). Moreover, significantly enriched terms in molecular function found for up-regulated IGs are consistent with their regulatory role, including rRNA methyltransferase and DNA-binding transcription repressor activities. In contrast, in the up-regulated MEGs the molecular function terms are highly enriched for transmembrane transporters and channel voltage activities (Figure 5D).

**FIGURE 6:**
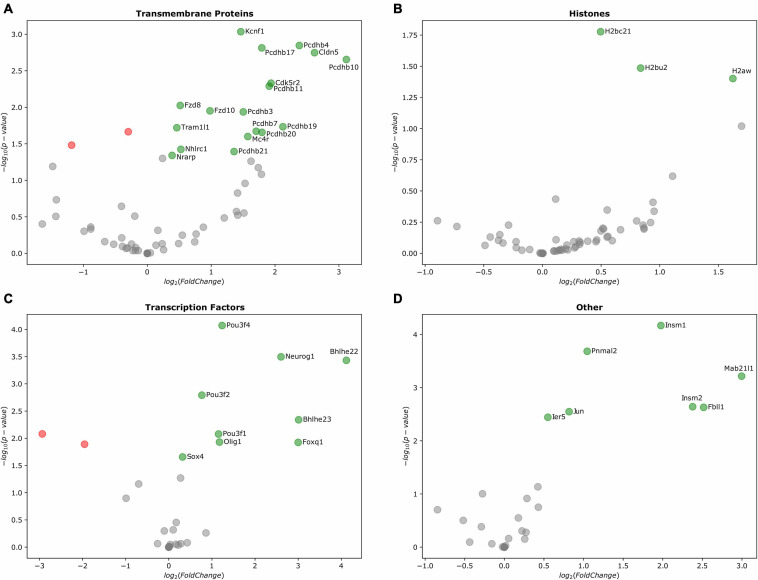
Differentially expressed IGs in the mouse embryonic telencephalon. Expression of genes grouped by their Pfam assignment, determined by their log2-fold change values. Up-regulated genes are highlighted in green, while downregulated genes are colored in red. **(A)** Gene expression of transmembrane proteins, **(B)** histones, **(C)** transcription factors, and **(D)** other protein families.

**FIGURE 7:**
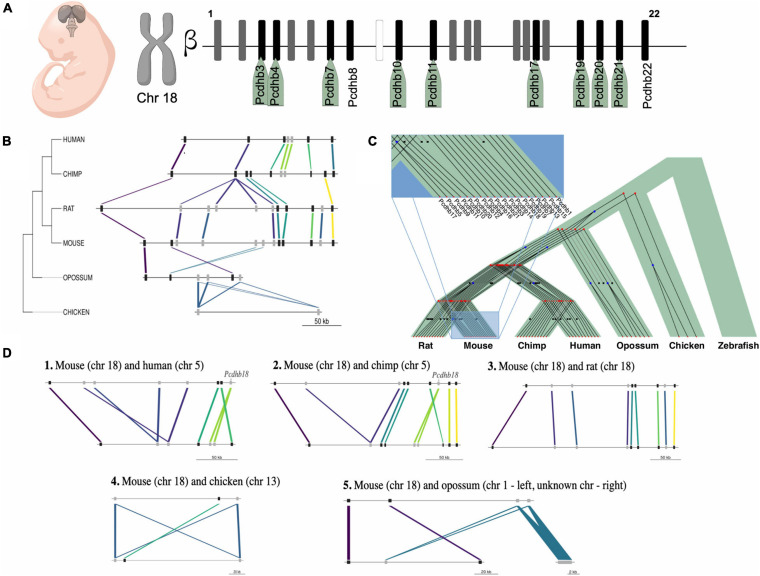
Expression and evolution analysis of the mouse cluster of β-protocadherins. **(A)** Cluster of the 22 β-protocadherins in the chromosome 18 depicting in black up-regulated genes (names highlighted in green) in telencephalon between embryonic stages 9.5 and 10.5, in gray expressed genes with no relative changes between stages, and in white not expressed genes; **(B)** Syntenic map of the approximately 300 kb β-protocadherin locus across five mammalian lineages and chicken as an outgroup. Colored lines depict orthology relationships across the phylogenetic tree. Genes connected by the same color belong to the same orthogroup identified by ProteinOrtho. Genes are shown as black squares for single-copy orthologs and gray squares for expanded genes; **(C)** Reconciliation tree of the protocadherin gene trees and the species tree. Each gene tree represents an orthogroup, internal nodes represent evolutionary events (blue squares represent duplications, red bullets represent speciations) and black crosses in leaves represent inferred gene loss events; **(D)** Pairwise syntenic comparisons of the mouse β-protocadherin locus to four mammalian genomes and chick highlighting the lineage-specific loss and expansions of the *Pcdhb18* gene.

From the IG transmembrane protein group, transcripts for *Tram1l1, Cdk5r2, Nrarp, Kcnf1, Fzd7, Fzd8, Fzd10*, and *Cldn5* were up-regulated. Strikingly, from the cluster of 22 β*-*protocadherins (pcdhbs), which contains 18 IGs, 9 of these were among those up-regulated in the E10.5 telencephalon (*Pcdhb3, Pcdhb4, Pcdhb7, Pcdhb10, Pcdhb11, Pcdhb17, Pcdhb19, Pcdhb20*, and *Pcdhb21*) (Figures 6A, 7A). It is important to note that all but one of all protocadherins of this cluster were expressed in the developing telencephalon. Our expression analysis additionally revealed up-regulation of *Olig1, Bhlhe22, Bhlhe23, Pou3f1, Pou3f2, Pou3f4, Foxq1*, and *Neurog1*, most of which are BHLH transcription factors crucial for the regulation of brain development and neuro-specific functions (Figure 6B). Moreover, regarding IGs within the histone group, *H2bc21, H2bu2, H2aw* were up-regulated during the mouse embryonic stages (Figure 6C). Finally, IGs from other groups with up-regulation were also observed (Figure 6D).

### The β-Protocadherin Gene Cluster Displays a High Degree of Syntenic Conservation Across Mammalian Genomes

To gain further insight on the evolutionary conservation of IGs with a functional role in telencephalon patterning, we studied the syntenic conservation of the ortholog genes of the 18 mouse IG β*-*protocadherins (Figure 7A) in our set of seven species. We determined that human, chimp, rat, opossum, and chick contain orthologs of the mouse single exon *pcdhb* genes, which are absent in zebrafish. Overall, all the orthologous genes of the cluster are located in a single locus in their respective genomes, with varying lengths ranging from ∼128 to 310 kb, displaying a few local inversions (Figure 7B). These results are consistent with previous studies that have explored the syntenic conservation of *pcdhb* genes across other vertebrate species (Noonan et al., 2004; Yu et al., 2008). Even though we found syntenic conservation of some members of the β*-*protocadherin cluster, we observed slight disruptions of the order of genes due to gene expansions, which can be either gene duplications or *de novo* formation. These gains are most notorious in the mammalian genomes (Figure 7B), suggesting gene expansion of the intronless β*-*protocadherins could be relevant for their role in neurogenesis, as well as other neuro-specific functions associated with the Wnt canonical pathway.

We looked in more detail at the evolutionary histories of β*-*protocadherins, by reconciling the gene trees of this gene cluster with the taxonomic species tree (Figure 7C). First, we observed that none of these genes is conserved in zebrafish. Moreover, some genes are gained in specific lineages, for example, *Pcdhb17* is only observed in mice and rats, while eight genes are shared across the mammals in the study. Three β-protocadherins appear to be shared among primates and the marsupial opossum, while only *Pcdhb7, Pcdhb15*, and *Pcdhb19* are shared between mouse and chick, and across other intermediate species, suggesting that these are the oldest β*-*protocadherins that give origin to the rest of them.

Then, by assessing syntenic conservation in a pair-wise fashion, we found relevant lineage-specific gene losses (Figure 7D). For instance, *Pcdhb18* is absent in rats while it is present in mice and duplicated in primates. This evidence suggests that the complexity of nervous system characteristic of mammals could also be associated with the duplication of single exon genes besides splicing-derived protein isoform diversity.

### Characterization and Functional Role of Post-translational Modifications in Mouse IG Proteins

**FIGURE 8:**
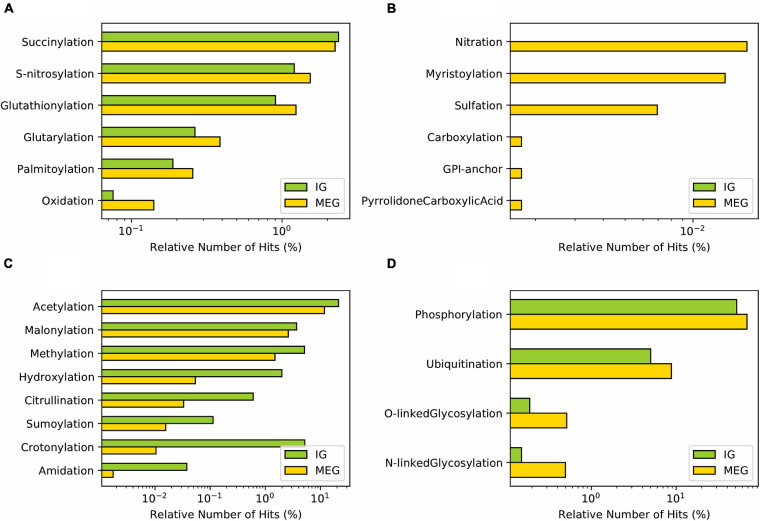
Distribution of post-translational modifications in mouse IG proteins compared to MEG proteins. **(A)** PTMs with similar distribution between IG and MEG proteins. **(B)** PTMs exclusive to MEGs. **(C)** PTMs predominant in IG, and **(D)** in MEG proteins.

In addition to alternative splicing, and mRNA editing, post-translational modifications (PTMs) constitute a defining factor of the complexity of proteomes by increasing structural and functional diversity of each proteoform, the set of multiple protein molecules encoded by one gene. Hence, protein PTMs have an essential role in protein structure-function, including activity, stability, folding, and turnover (Uversky, 2015). Since IGs fit the “one gene—one protein” concept we aimed to determine whether PTMs represent exclusive mechanisms of regulation for these genes. In our analysis, we observed that Succinylation and S-nitrosylation had similar prevalence in IG and MEG groups. These were followed with much lower frequency by Glutathionylation, Glutarylation, Palmitoylation, and Oxidation (Figure 8A). In contrast, PTMs with a unique presence in MEGs were Nitration, Myristoylation, Sulfation, Carboxylation, GPI-anchor, and Pyrrolidone carboxylic acid (Figure 8B).

Notably, in accordance with their protein assignment, a differential enrichment for IG proteins was observed of Acetylation, Crotonylation, Methylation, Malonylation, and Hydroxylation (Figure 8C) which are characteristic features of the core histones. Other PTMs of IG proteins, albeit at lower frequency, were Citrullination, Sumoylation, and Amidation (Figure 8C). The complementary group of PTMs more enriched in MEG proteins included Phosphorylation, followed by O-linked Glycosylation, Ubiquitination, and N-linked Glycosylation (Figure 8D). PTMs classified based on the modification-enabled functionality for membrane localization such as Myristoylation and GPI-anchor were found to be more frequent for MEGs (Figure 8D).

### Functional Assignment of IGs as Microproteins

The regulation of multidomain proteins at the post-translational level can be mediated by microproteins (miPs) (Staudt and Wenkel, 2011) which are small proteins containing a single domain that form heterodimers with their targets and exert dominant-negative regulatory effects (de Klein et al., 2015; Eguen et al., 2015). In *Eukarya*, microproteins have been found to have a remarkable influence on diverse biological processes.

Aware of the differential occurrence of PTMs on IG proteins, the DNA binding repressor activity molecular function of up-regulated IGs during mouse brain development, and due to the remarkable role of miPs, we assessed whether this group of genes encoded proteins fitting the miP definition. Characteristic features of miPs are the short length of their primary structure, a homodimer domain, and negative modulating activity of protein multi-complexes. Our first approach was to analyze the peptide length of IG and MEG proteins. The highest length-frequency for IG peptides was in the range of 200–400 amino acids, compared to that of MEG peptides which was 300–500. Then, using the miPfinder tool (Straub and Wenkel, 2017), we identified the following IGs as microprotein candidates: the BHLH transcription factors *Bhlha9*, *Msg1*, *Ferd3l*, *Bhlhe23*, and *Ascl5* (*e*-value 4.6E–30), as well as the histones *H1f0, H1f1, Hils1, H1f2 H1f3, H1f4, H1f5, H1f6*, and *H1f10* (*e*-value 7.4E–09), corresponding to the H1 linker histone group.

### Conservation and Evolution of IGs Across Vertebrata

**FIGURE 9:**
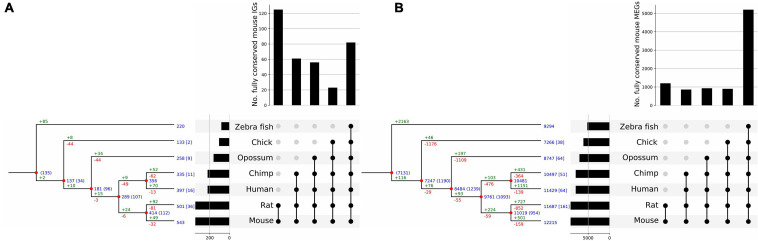
Clusterization of conserved mouse IG and MEG orthologs. Reconciliation trees representing the evolutionary history of mouse IG **(A)**, and MEG **(B)** gene families. Blue numbers at each internal node represent the number of ancestral genes found in that clade that were inherited by an older ancestor. Blue numbers in brackets represent ancestral genes that might be generated at that evolutionary point, since they are not found in an outgroup of the clade. Green numbers represent gene gains due to duplication events and red numbers represent gene losses. Numbers at the tip of the branches represent the number of orthologous genes in other species, and numbers in square brackets represent the number of out-paralogs (genes that arose via duplication before speciation) in other species in the study. Orthologous genes are grouped by age determined by the clade they are conserved in; the histogram on top of the upset plot shows the number of genes that are specific to each clade; the histogram to the left of the upset plot represents the number of mouse orthologs for each species. Moreover, the bar for mouse shows the number of IGs for which an ortholog in another species was found.

To infer the evolutionary age of genes we implemented a bioinformatics method to assess the extent and patterns of distribution of each gene’s orthologs and paralogs in different species. The rationale of this approach is that widespread conservation of the orthologs of a gene in the different vertebrate taxa is an indication of old age for that particular gene. This approach allowed us to determine the conservation of IGs across 7 genomes, as well as to identify species-specific mouse IGs (Figure 9). In this analysis, we found that 543 out of the 1,116 mouse IGs have orthologs in at least one of the other species. For the mammalian genomes, we found 442 genes conserved as IGs out of 501 orthologs in the rat genome, 335 orthologs in chimp with 250 conserved as IGs, 397 with 262 IGs in human, and 258 with 167 IGs in opossum (Table 1). Meanwhile, we found 133 orthologs in chick with 78 conserved as IGs, and 220 in zebrafish with 91 conserved as IGs (Table 1). We also identified out-paralogs of mouse IGs (genes that arose via duplication before a speciation) that are conserved in the other species: 36 in rat, 16 in human, 11 in chimp, 9 in opossum, 2 in chick and none in zebrafish. Finally, we identified 573 IGs with no orthologs in the other species, suggesting that these are species-specific mouse IGs.

**TABLE 1:** Summary of mouse IG orthologs in selected genomes.

Genome	IGs	MEGs	Others	Total
Zebrafish	91	90	39	220
Chick	78	55	0	133
Opposum	167	87	4	258
Chimp	250	59	26	335
Human	262	97	38	397
Rat	442	57	2	501

Overall, we found that 70% of the IG orthologs are IGs as well, and 30% are MEGs (Table 1). As for MEGs (Figure 9), less than 5% of their orthologs are IGs and the rest are MEGs (Tables 1, 2). As expected, due to its evolutionary closeness with the mouse, the genome with the highest conservation in gene architecture is the rat, with approximately 88% of conserved IGs, while the largest difference was found for the zebrafish genome with only 41% of conserved IGs. Furthermore, at the superfamily level as identified by SUPERFAMILY, we found that 24% of the IG superfamilies are conserved as IG-only, while 76% are predominantly IGs but contain at least one MEG ortholog in another species. Similarly, for MEG superfamilies, 35% were conserved as MEG-only, while 65% were predominantly MEGs with at least one IG ortholog. Hence, these analyses revealed that most of the IGs identified in the mouse genome remained with this genetic structure in other species thus supporting their high conservation across vertebrate genomes.

**TABLE 2:** Summary of mouse MEG orthologs in selected genomes.

Genome	IGs	MEGs	Others	Total
Zebrafish	108	7,602	1,584	9,294
Chick	122	7,137	7	7,266
Opposum	159	10,304	966	11,429
Chimp	523	8,040	184	8,747
Human	603	9,520	374	10,497
Rat	1,171	10,457	59	11,687

From the previous analysis we clusterized IGs and MEGs into five age-groups named by the taxonomic category that includes all the species of each group and thus represents the most recent common ancestor (MRCA) of each ortholog as inferred from the extant species analyzed. These groups were: *Vertebrata*, *Tetrapoda*, *Theria*, *Eutheria*, and *Muridae* (Figure 9). From the reconstruction of the evolutionary history of the mouse IGs, our results revealed that their conservation is more marked in the *Muridae* as it contains the largest number of orthologs common to its members (Figure 9A) followed in abundance by *Vertebrata*. This indicates that a large number of IGs are sufficiently old to have orthologs in all the vertebrates analyzed, and that the clades that include the closest relatives to mice have increasing IG ortholog abundance. In contrast, the highest conservation of MEGs in gene numbers is among *Vertebrata* thereby revealing a much older age than that of *Muridae* IGs (Figure 9B). For both IG and MEG orthologs, the number of paralog-related genes increases with gene age consistently with the rate of duplication of the edges of each clade (Figure 9). Moreover, a significant number of in-paralogous genes in the zebrafish genome, generated via duplication after speciation, have an ortholog in the mouse genome.

**FIGURE 10:**
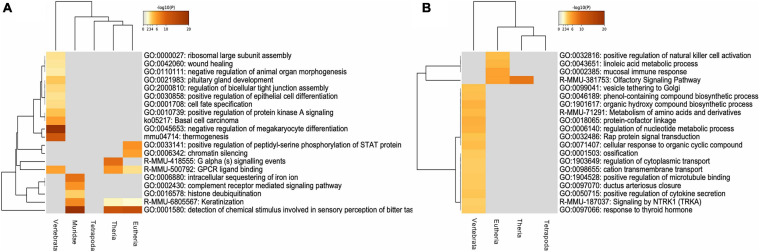
Functional enrichment of conserved mouse IG and MEG orthologs. **(A)** Gene ontology enrichment and pathways for mouse IG conserved as IG in the selected species, orthologs are clustered regarding their “age” group. **(B)** Gene ontology enrichment and pathways for a sample of 1,116 mouse MEGs conserved as MEG in the selected species, orthologs are clustered regarding their “age” group. The color key from yellow to brown indicates high to low *p*-values, respectively.

With the purpose of determining whether there was a differential functional enrichment of IGs according to their evolutionary age, we analyzed the enrichment of molecular pathway GO terms in both IGs and MEGs. In agreement with a specialized role of IGs, our results show that IG and MEG orthologs are involved in different biological pathways although some shared pathways were detected as well (Figure 10). Conserved IG proteins with the MRCA among *Vertebrata* are histones highly enriched in negative regulation of megakaryocyte differentiation (–log10, –20.82). Other orthologs conserved to this group are linked in a lower level to thermogenesis, basal cell carcinoma, positive regulation of protein kinase A signaling, ribosomal large subunit assembly, wound healing, and vascular process in the circulatory system, platelet aggregation and development process such as cell-fate specification, negative regulation of animal organ morphogenesis, pituitary gland development, and regulation of bicellular tight junction assembly (–log10, –9.33 > −2.81). For *Theria* we found G alpha signaling events (–log10, –8.86), while in the *Eutheria* group peptidyl-serine phosphorylation of STAT protein, and chromatin silencing were enriched (–log10, –6.47;–5.69). Noticeably, the most recent genes which belong to the *Muridae* group are exclusively enriched in intracellular sequestering of iron, complement receptor-mediated signaling pathway, and histone deubiquitination (–log10, –8.64 > −3.63).

Our analysis also identified IG proteins with enriched pathways shared among the various age groups. Detection of chemical stimulus involved in sensory perception of bitter taste, and keratinization are GO terms shared among *Muridae* (–log10, –22.91;–7.07), *Eutheria* (–log10, –13.36;–2.10) and *Theria* (–log10, –12.37;–2.39). Meanwhile, GPCR ligand binding is shared among *Vertebrata* (–log10, –5.59), *Theria* (–log10, –6.11), and *Eutheria* (–log10, –3.05) (Figure 10A).

Then we focused on determining the conservation of the biological role of IG orthologs among the different genomes. GO terms that are highly enriched in the seven genomes analyzed were detection of chemical stimulus involved in sensory perception of smell, organic substance metabolism, DNA packaging, signaling, multicellular organismal process, cell communication, transport, and localization, while GO molecular function terms enriched are olfactory receptor activity, Wnt protein binding, odorant binding, protein binding, catalytic, and molecular transducer activity.

## Discussion

The mechanism of alternative splicing is a pivotal contributor to the diversity of proteins and the functional complexity of eukaryotic genomes. Intron-containing genes are capable of generating multiple protein isoforms by this process by which exons can be removed, lengthened, or shortened (Sakharkar et al., 2005b). In contrast, protein coding genes lacking introns produce a single peptide of predictable sequence which may undergo postranslational fine tuning. The availability of detailed annotation of sequenced genomes for many organisms contributes toward a better understanding of their structure which has been shaped by flexible evolutionary pressure (Bult et al., 2019). Studying the evolutionary dynamics of exon-intron patterns at the genomic level is likely to shed light on their role in genome structure and gene architecture.

To further our insight into the structure and the evolution of mouse IGs, we examined their function, differential expression in the developing brain, the signatures for post-translational modifications of their encoded proteins, their potential as modulators of multiprotein complexes, as well as their evolutionary dynamics in comparison to their orthologs in other vertebrates.

Our work revealed that, in accordance with previous studies, IGs and MEGs appear to specialize in different functions which is supported by their enrichment in distinct biological pathways and differential abundance of post-translational modifications. As an additional indication of this specialization, IGs that are up-regulated during the development of the mouse telencephalon, are associated with specific developmental programs in this structure and display a functional enrichment profile that differs from that of up-regulated MEGs. Moreover, mouse IGs, some of which fit the criteria to be defined as regulatory microproteins, appear to be of more recent origin than MEGs in vertebrates. Consistent with this notion, about half of IGs do not appear to have orthologs in other genomes thus suggesting a relevant role in mouse evolution. The synteny of the β*-*protocadherins, however, points out to a mammalian conserved function of these IGs although some species-specific changes in this gene cluster were observed for the various species analyzed.

### Functional Assignment of IGs Highlights Prevalent and Unique Biological Roles

In the mouse genome, coding genes are predominantly of MEG type^11^ (80% of a total of 22,481). However, a considerable number of IGs are present in this genome, and the conservation of this fraction has been reported for other mammalian genomes (Sakharkar et al., 2006).

Our comparative analysis of the types of proteins encoded by IGs and MEGs revealed that these two populations have very divergent functional profiles. The most abundant types of proteins found among the former were the chromatin components histones and centromere proteins, transmembrane proteins of the G-protein coupled receptor family 1 and cadherins, and transcription factors containing BTB, forkhead, and HLH domains. In contrast, among MEGs, the most abundant proteins were those containing zinc finger, Pkinase (Protein-kinase), and PH (Pleckstrin homology) domains. These observations are consistent with previous findings that IGs are highly enriched in GPCRs, and seven transmembrane domain proteins and reveal a functional divergence between IGs and MEGs likely to be the result of differential evolutionary constraints (Sakharkar et al., 2006).

Among the transmembrane IG proteins, vomeronasal and taste receptors stand out as the most abundant. These proteins play a highly relevant role in chemoreception which is the most salient means of the interaction of *Muridae* with their environment, with conspecifics, with potential prey or predators. These findings suggest that some olfaction and taste receptors are required to be constantly transcribed in an efficient and rapid process, which may be a factor that favors their overrepresentation among IGs. Moreover, the taste receptor cells of vertebrates are continually renewed throughout the organism’s life which suggests a high demand for the housekeeping expression of these genes.

A considerable enrichment was also observed among IGs of BTB, Forkhead, HLH, HMG-Box transcription factors, and the chromatin components core histones H2AC, CENP-TC, and the histone Linker cluster. Thus, this indicates that IGs are playing also an important role in packaging, transcription and chromatin assembly.

Altogether, these results suggest that intronless genes have specialized roles, and a strong link to gene expression regulation and chromatin structure. Overall, our results suggest that IG proteins have specialized, prevalent, and unique biological roles.

### Differential Expression of IGs in Telencephalon Development, Key Genes for Wnt Signaling

Embryonic development relies on the complex interplay of fundamental cellular processes, including proliferation, differentiation, and apoptosis. Regulation of these events is essential for the establishment of structures and organ development. The formation of the telencephalic architecture results from the interaction of the signaling centers located on the edges of the pallium. In this process, the Wnt signaling pathway plays an essential role in the dorsomedial pattern, where signals from the cortical hem direct the morphogenesis of the hippocampus, the corpus callosum, and the generation of migratory Cajal-Retzius cells. From the lateral pallium, the anti-hem signals, EGF, FGF, Frizzled, and Sfrp determine the development of the olfactory cortex.

In a previous study, we highlighted the up-regulation of Wnt signaling genes of the canonical pathway in the early stages of the developing telencephalon in mice (Muley et al., 2020). The main receptors of the Wnt/beta-catenin signaling pathway are Frizzled domain proteins (Fzd), a family of seven-transmembrane G-protein coupled receptors that also possess a large extracellular cysteine-rich domain.

In this work, we found *Fzd7, Fzd8*, and *Fzd10* among the IG transmembrane proteins that were developmentally regulated in the embryonic telencephalon, as well as a group of 11 IGs of the protocadherin β-cluster. Previous studies have described *Fzd8* as an essential receptor of the Wnt pathway implicated in brain development and size (Boyd et al., 2015). This gene is also highly expressed in two human cancer cell lines, indicating that it may play a role in tumorigenesis (Li et al., 2017; Murillo-Garzón et al., 2018). *Fzd10* functions in the canonical Wnt/beta-catenin signaling pathway which may be involved in signal transduction during tissue morphogenesis (Wang et al., 2005). In keeping with this, protocadherins have also been described as regulators in the Wnt signaling pathway (Mah and Weiner, 2017). More specifically, protocadherins of the β-cluster, along with those of the α and γ clusters, act cooperatively in mice in olfactory-axon targeting, in the formation of diverse neural circuits, and in neuronal survival (Hasegawa et al., 2016, 2017). These functions, however, correspond to developmental stages that occur later than the one addressed in this study. Hence, our striking finding that half of the 22 β-protocadherins are up-regulated along with Wnt receptors during the development of the telencephalon, suggest that this group of mostly IGs has a differential function thus far unknown related to Wnt signaling at this early stage. Consistent with this idea, the Wnt binding molecular function GO term is one of the most conserved among IGs in the genomes analyzed in this study.

Our expression analysis additionally revealed up-regulation of *Olig1, Bhlhe22, Bhlhe23, Pou3f1, Pou3f2, Pou3f4, Foxq1*, and *Neurog1*. Notably, this represents the up-regulation of three of the four members of the *Pou3* class of transcription factors present in mouse. The *Pou* genes encode a broad family of 6 classes (*Pou1f*–*Pou6f*) which are involved in developmental processes, mainly cell fate determination and differentiation (Tantin, 2013). Among those, the four members of the *Pou3f* class are preferentially expressed in ectodermal derivatives such as the developing mammalian nervous system (Bally-Cuif and Hammerschmidt, 2003). The human *Pou3f3* is an intronless gene also named *Brain-1*, which is a well-known transcription factor involved in the development of the central nervous system and its variant alleles have been associated with intellectual disability and language neurodevelopmental disorders (Snijders Blok et al., 2019). Furthermore, an important role of *Neurog1* is as a promoter of proliferation or neuronal differentiation, while *Olig1* is involved in the generation and maturation of specific neural cells during the development of the spinal cord (Qi et al., 2016; Song et al., 2017). *Bhlhe22* and *Bhlhe23* in turn, are among those that were up-regulated the most in mice during telencephalon development. In humans, *Bhlhe22* has been identified as a highly methylated gene in endometrial cancer with potential epigenetic biomarkers in cervical scrapings (Liew et al., 2019), while *Bhlhe23* has been linked to mammalian retinal development (Woods et al., 2018).

Finally, among the histone group, *H2bc21 (Hist2h2be), H2bu2 (Hist3h2ba), H2aw (Hist3h2a)* were also up-regulated. The *H2b* histone family members are responsible for the chromosomal fiber nucleosome structure in eukaryotes. *H2bc21/Hist2h2be* has been described in mouse as expressed in olfactory epithelium, while *H2bu2 (Hist3h2ba)* in neocortex and lens of camera type-eye, and *H2aw (Hist3h2a)* in retina^12^. In humans, *Hist2h2be* is a hub gene related to poor prognosis in rhabdomyosarcoma tumors in pediatric patients (Li et al., 2019).

Summarizing, IGs appear to play crucial roles in the mouse telencephalon involved in gliogenesis, eye, and sensory organ development, canonical Wnt signaling, nucleosome organization, and have molecular regulatory roles. Therefore, in accordance with the functional assignment, our expression analysis supports that IGs play a critical role during mammalian brain development.

### IG Proteins in the Histone Category Have Unique Signatures and Undergo Specific PTMs

In agreement with the link of IGs to chromatin structure found in this and previous works, we also identified unique and highly represented PTM signatures in the histone protein category. Proteins encoded by mouse IGs have enriched signatures for histone *H2A* and *H2B*, characteristic of key core histones involved in chromatin structure in eukaryotic cells, as well as linker histone *H1/H5* and the *CENB-type HTH*. In addition to the identification of exclusive signatures, we compared potential regulatory mechanisms of IG and MEG PTMs. Although the variability of PTMs is high, these modifications are typically very specific and, altogether, 300 types are known to occur in proteins (Witze et al., 2007). Among all PTMs, we found that the most abundant (Phosphorylation and Acetylation) are differentially represented among IG and MEG proteins.

As it could be expected from the observed enrichment in chromatin remodeling protein domains, our results show that proteins encoded by IGs undergo specific PTMs for histones such as crotonylation, methylation, sumoylation, citrullination, and sumoylation. However, our results suggest that IG-encoded histones have high specificity for Lysine-crotonylation, which is a recently identified post-translational modification associated with active promoters to directly stimulate transcription. Moreover, PTMs with changes in the physicochemical properties of amino acids like citrullination and amidation, are a characteristic feature highly enriched in IG proteins.

### Potential Role of IGs as miPs in Neural Development and Function

When we assessed the potential role of IGs as microproteins we found proteins with strong potential to be modulators of multi-protein complexes. The targets or microproteins are mostly transcription factors that bind DNA as dimers. In this study, we found potential miPs encoded by intronless genes that are *bHLH* transcription factors with a regulatory role during critical events such as neural development and function. For example, *Ferd3l*, an evolutionarily conserved *bHLH* protein, is expressed in the developing central nervous system and functions as a transcriptional inhibitor. Other examples are *bHLHe23*, a transcriptional regulator in the pancreas and brain that marks the dimesencephalic boundary (Bramblett et al., 2002), *Bhlha9* a regulator of apical ectodermal ridge formation during limb development (Kataoka et al., 2018), *Msg1* which is predominantly expressed in nascent mesoderm, the heart tube, limb bud, and sclerotome during mouse embryogenesis (Dunwoodie et al., 1998), and *Ascl5* member of the ASCL family of proneural transcription factors that control the development of the nervous system, particularly neuroblast cell fate determination (Guillemot et al., 1993). Moreover, its potential role in tumorigenesis has been described with up-regulation in lung cancer and down-regulation in brain tumors such as glioblastoma, anaplastic oligoastrocytoma, anaplastic oligodendroglioma, and oligodendroglioma (Wang et al., 2017). Additionally, consistent with the potential role in the development of IG-encoded miPs, we identified members of the *H1* linker histone group that fit the criteria to be classified as miPs. These histone proteins belong to a complex family with distinct specificity for tissues, developmental stages, and organisms in which they are expressed (Izzo et al., 2008).

### Patterns of Evolution of IGs Differ From Those of MEGs in Vertebrates

According to earlier studies that reported the high conservation of mouse IG orthologs among other eukaryotic genomes, our analysis across seven species belonging to three classes of vertebrates revealed that the most numerous orthologs in each species were also IGs. This high rate of genetic structure conservation has been previously associated with their essential role in cell housekeeping functions, particularly those functionally pivotal proteins involved in molecular and biological roles such as transcription, translation, energy metabolism, amino-acid biosynthesis, and binding, which must be highly conserved (Sakharkar et al., 2006). Individually, eutherians (rat, chimp, human) are the species with the most orthologs. Moreover, *Muridae*, the clade that includes the mouse and rat, has the largest number of conserved orthologs and this number decreases as the clades gradually include the more distantly related species. Moreover, a distinctly large number was also found common to all species analyzed (*Vertebrata*), which is consistent with previous findings that identified functional and evolutionary conservation of eukaryotic IGs with highly distant genomes such as bacteria (Sakharkar et al., 2006). The higher abundance of conserved IG orthologs among species more closely related to mouse and lower in groups including more distant species, could be the result of the gradual loss of IG orthologs during the divergence of the diverse vertebrate branches. This abundance, however, could also be due to an increased rate of IG generation among mammals. Evidence supporting the latter possibility comes from our finding that in stark contrast, an increase of conserved MEG orthologs among species more closely related to mouse was not observed.

The clade *Vertebrata* contains the older IGs that are involved in diverse functions, among which nucleosome structure stands out. Histone IGs are conserved among all species, with some losses in opossum and chick. As histones are basic proteins known to be conserved across eukaryotes, it is not surprising that they are found to be some of the oldest IGs. In some cases, histones are conserved in the genome as clusters, and in some others, they appear to have been generated in a specific lineage, due to multiple gene duplications. *Muridae*, in contrast to *Vertebrata*, contains the more recent IGs which are involved in keratinization, GPCRs, and chemodetection by GPCRs. We can conclude that *Muridae* IGs are younger and have more specific functions, whereas IGs conserved in all vertebrates are more ancient and have more general or basic functions, as expected due to the high conservation of sequence and function among vertebrates.

Gene duplication is an important mechanism for the acquisition of new genes, frequently providing specialized or new gene functions (Magadum et al., 2013). Known mechanisms of gene duplication include retroposition, tandem duplication, and genome duplication (Pan and Zhang, 2008). Our analysis shows that the vast majority (48%) of one exon genes in the mouse genome are a consequence of retroposition. Moreover, regarding the duplication events, we found clear examples of IG tandem repeat cluster organization. For example, the syntenic conservation of the tandem cluster of β-protocadherins and their neural tissue-specific expression suggest that some aspects of the nervous system characteristic of mammals could be associated with the duplication of intronless genes, such as olfactory-axon targeting, the formation of neural circuits, neuronal survival, or neurite self-avoidance during development (Dennis et al., 2016; Hasegawa et al., 2017; Brasch et al., 2019). Similar to the single exon β-cluster of protocadherins, we also observed that IG histones in the mouse genome are present as tandem families with a tendency to cluster in their chromosome organization. An example of this is the *H2A* histone family member *L1J*, a family of ten IG members in the mouse X chromosome, with only one ortholog (*H2AL1RP*) in human and one (*H2A-beta*) in opossum. Almost all of the tandem repeat genes have parallel transcription orientation, which means they are encoded on the same strand.

The disrupted gene structure of most eukaryotic genes has led to a long-lasting debate regarding the origin of introns. The “exon theory of genes” also known as “introns-early,” proposed the presence of introns in prokaryotic primordial genes (Gilbert, 1987; Roy and Gilbert, 2006), while in the “insertional theory of introns” or “introns-late theory,” introns are a eukaryotic innovation (Doolittle and Stoltzfus, 1993; Stoltzfus, 1994; Stoltzfus et al., 1994; Mattick, 1994; Logsdon, 1998). Recent genomic evidence supports a view that combines aspects of both theories but still placing the invasion of eukaryotic genes by introns at the emergence of eukaryotic cells (Koonin, 2007). In accordance with this combined view, the comparison of mouse intron-bearing and intron-lacking IG orthologs among the analyzed organisms, suggests that IGs are more recent than MEGs. This is also consistent with findings that have revealed that intron-exon gene structure is highly stable among vertebrates and that individual intron losses outnumber intron gains in diverse vertebrate lineages (Roy et al., 2003; Coulombe-Huntington and Majewski, 2007; Loh et al., 2008; Venkatesh et al., 2014). This evolutionary stability also suggests that the observed increase in IG abundance in *Muridae* is due to gene duplication of IGs rather than intron loss of MEG orthologs. Our findings, however, also revealed some interesting gene superfamilies of a few members each, containing only IGs which were restricted to one or two species. These IGs are also likely to have been generated recently in the branches leading to the species analyzed herein but the mechanism involved remains to be studied further.

The present study aimed to identify the conservation of the role of intronless genes in mammals and other vertebrate genomes. A comprehensive understanding of their biological function is essential to compare and contrast their evolution with that of intron-containing genes. Hence, we studied the complex regulatory role of intronless genes and their conservation in cellular environments using computational functional assignment, gene expression analysis, and evolutionary reconstruction. First, we determined that the functions associated with IGs are very different from those associated with MEGs. Expression analysis of the developing telencephalon also revealed specific up-regulation of IGs that encode genes involved in Wnt signaling, *bHLH* and *Pou* transcription factors, as well as chromatin proteins. Among Wnt signaling-related proteins, it was striking to detect up-regulation of half of all protocadherins of the β-cluster. Moreover, some IG transcription factors meet the criteria to be considered microproteins and thus appear to have modulatory properties of protein complex formation. Overall, our results highlight a role for IGs as essential modulators of diverse biological processes as pivotal as cortical development, chemosensory functions, chromatin condensation, and gene silencing. In fact, specific modifications of IG proteins indicate that their regulatory roles extend to the post-translational level. Notably, some of the IGs highlighted in this study also have potential clinical relevance in humans. For example, *Fzd8* and *pcdhs* which are associated to Wnt signaling, an evolutionarily conserved regulatory pathway related to cell fate determination and proliferation during development, have also been identified as part of a key mechanism in cancer biology. Other IGs discussed in this study and linked with cancer and neurodevelopmental disorders were *Pou3f3, bHLHE22, ASCL5*, and *Hist2h2be.*

Furthermore, the analysis of the evolutionary patterns of IGs revealed a large fraction of genes that appear to be of more recent generation as compared to the older and more conserved MEGs. Overall, this analysis reveals specific functions of IGs that distinguish them from MEGs and therefore strengthen the notion suggested by previous observations that these two groups are under differential evolutionary constraints.

## Data Availability Statement

Publicly available datasets were analyzed in this study. This data can be found here: https://data.mendeley.com/datasets/rdt5757cbw/1.

## Author Contributions

KA-P: project design, performed data collection, manuscript writing, proofreading, carried out bioinformatic analyses, prepared figures, and their interpretation. JR-R: expertise in bioinformatic analysis methods, performed evolutionary reconstruction, prepared figures. GH-O: literature search, bioinformatic analyses, and prepared figures. DV: writing, bioinformatic analysis, and prepared figures. ED-V: writing, bioinformatic analysis, and prepared figures. AG-G: expertise in data analysis methods for the API-REST and data collection, prepared figures. VM: proofreading, performed DEG analysis, prepared figures. AV-E: supervised the study, provided advice on the research strategy, and participated in manuscript writing. MH-R: co-director of the study and project development, performed bioinformatic analysis and interpretation, writing, and proofreading. All authors contributed to the article and approved the submitted version.

## Conflict of Interest

The authors declare that the research was conducted in the absence of any commercial or financial relationships that could be construed as a potential conflict of interest.

## Footnotes

1 https://github.com/GEmilioHO/intronless_genes

2 http://rest.ensembl.org

3 http://www.nextgenbioinformatics.org/IntronDB/

4 http://supfam.org/

5 https://pfam.xfam.org/

6 https://prosite.expasy.org/

7 http://metascape.org/

8 http://amigo.geneontology.org/amigo/landing

9 http://dbptm.mbc.nctu.edu.tw

10 https://github.com/DaStraub/miPFinder

11 https://www.ensembl.org/index.html

12 https://bgee.org/

## Supplementary Material

Supplementary Code https://github.com/GEmilioHO/intronless_genes.

Supplementary Tool https://gitlab.com/jarr.tecn/revolutionh-tl.

Supplementary Figure 1 Prevalence of intronless protein-coding genes among single-exon genes in the mouse genome. All mouse genes having one exon are classified regarding their gene biotype, proportion of protein-coding intronless genes (IGs) is highlighted in pink.

Supplementary Figure 2 Enrichment of SUPERFAMILY assignments of mouse IG and MEG proteins. **(A)** Enriched scop families in IG proteins: The scop families with the largest gene ratios are plotted in order of gene ratio. The size of the dots represents the number of genes in the significant background list associated with the scop family, while the color of the dots represents the adjusted p-values, **(B)** Enriched scop families in MEG proteins: The scop families with the largest gene ratios are plotted in order of gene ratio. The size of the dots represents the number of genes in the significant background list associated with the scop family, while the color of the dots represents the p-adjusted values.
