# Supplementary figures and images for: Evolutionary Perspective and Expression Analysis of Intronless Genes Highlight the Conservation of Their Regulatory Role
Source: Front Genet. 2021 Jul 9;12:654256. doi: 10.3389/fgene.2021.654256 (PMC8302217; doi:10.3389/fgene.2021.654256)

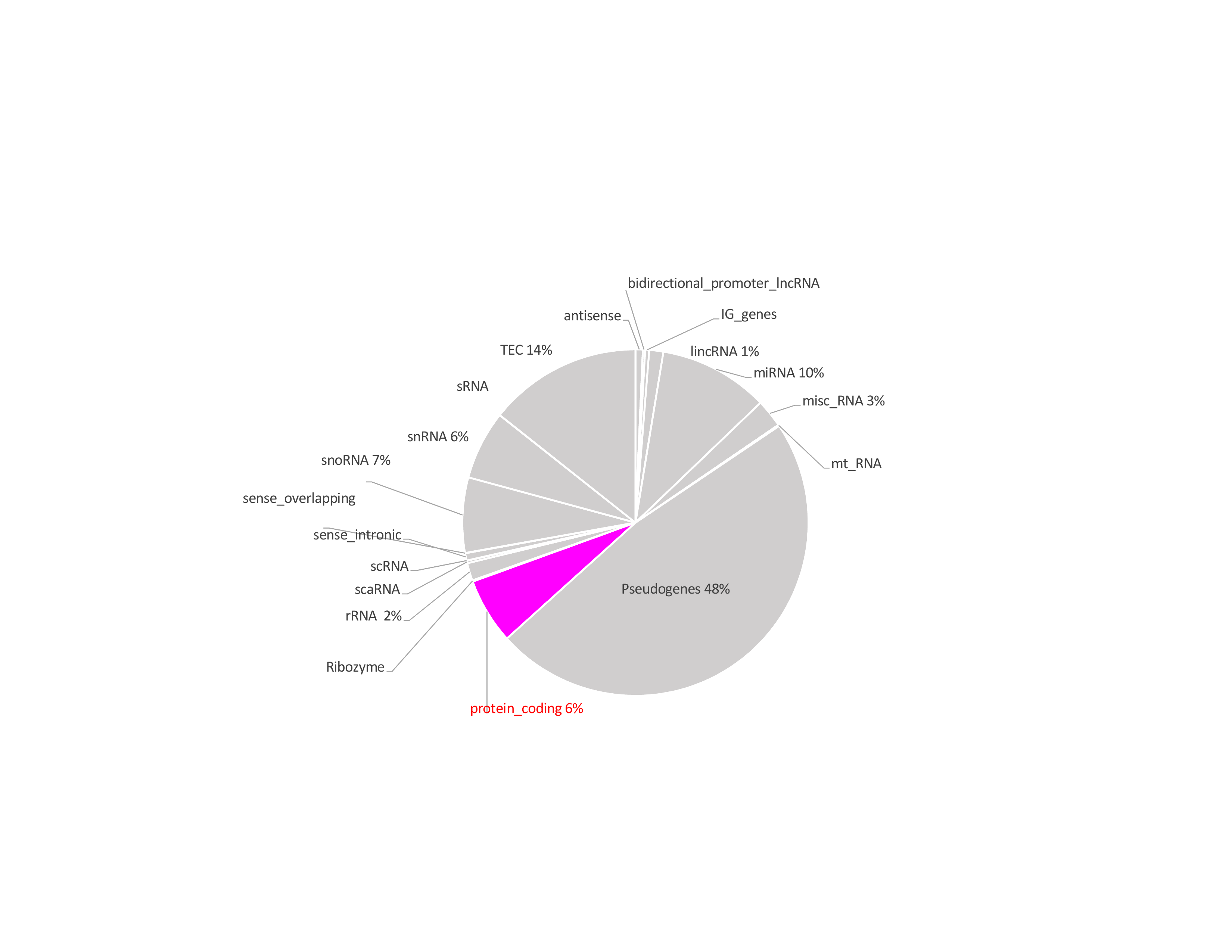

Supplement: Supplementary Figure 1 — Prevalence of intronless protein-coding genes among single-exon genes in the mouse genome. All mouse genes having one exon are classified regarding their gene biotype, proportion of protein-coding intronless genes (IGs) is highlighted in pink. [file Image_1.TIFF]

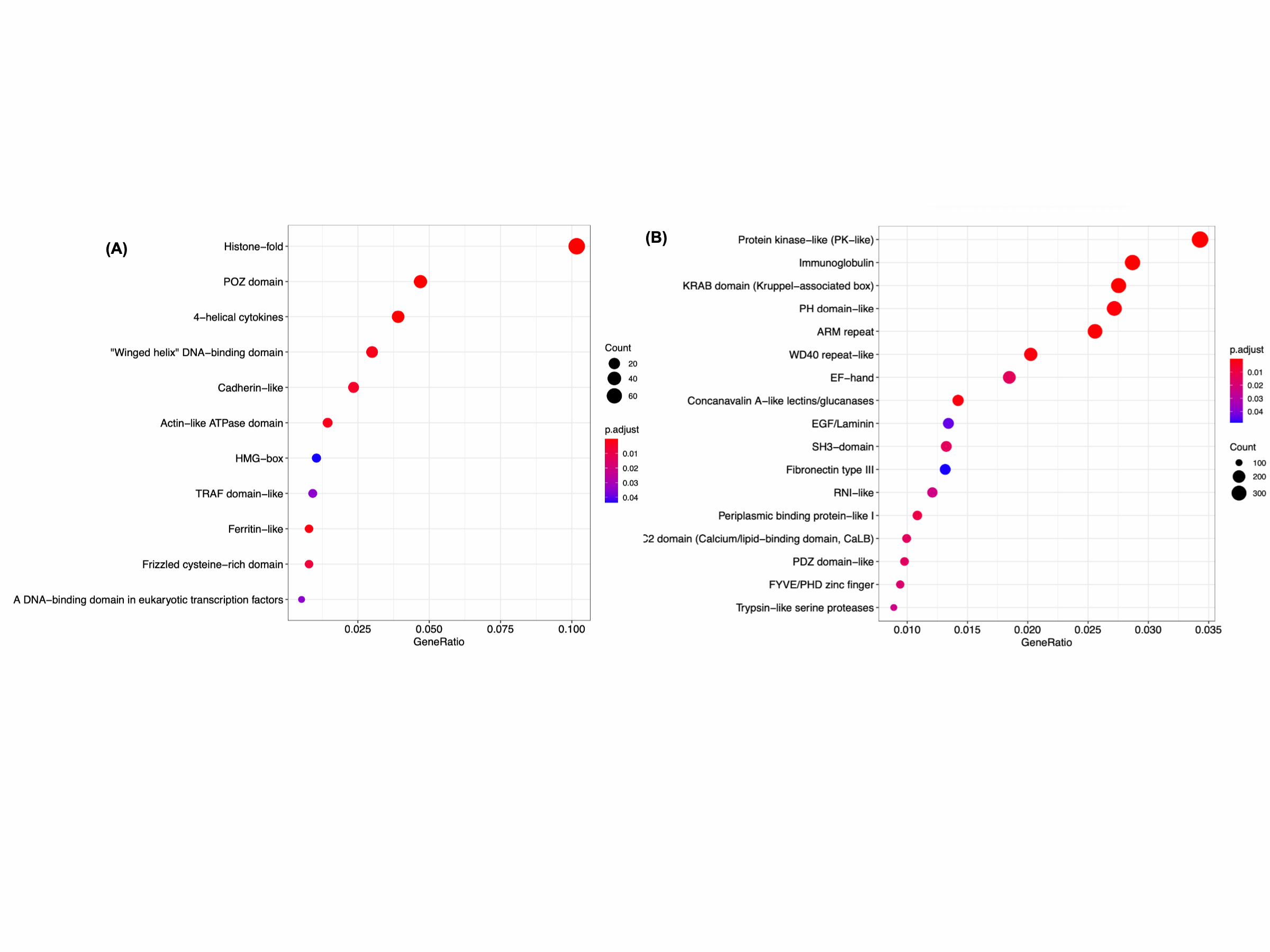

Supplement: Supplementary Figure 2 — Enrichment of SUPERFAMILY assignments of mouse IG and MEG proteins. (A) Enriched scop families in IG proteins: The scop families with the largest gene ratios are plotted in order of gene ratio. The size of the dots represents the number of genes in the significant background list associated with the scop family, while the color of the dots represents the adjusted p-values, (B) Enriched scop families in MEG proteins: The scop families with the largest gene ratios are plotted in order of gene ratio. The size of the dots represents the number of genes in the significant background list associated with the scop family, while the color of the dots represents the p-adjusted values. [file Image_2.TIFF]
